# Current Perspectives of Telomerase Structure and Function in Eukaryotes with Emerging Views on Telomerase in Human Parasites

**DOI:** 10.3390/ijms19020333

**Published:** 2018-01-24

**Authors:** Abhishek Dey, Kausik Chakrabarti

**Affiliations:** Department of Biological Sciences, The University of North Carolina at Charlotte, 9201 University City Boulevard, Charlotte, NC 28223, USA; adey2@uncc.edu

**Keywords:** telomerase, telomere, TER, TERT, parasite, *Trypanosoma brucei*, *Plasmodium falciparum*, RNA, RNP, DNA repair, cellular senescence, cancer, ageing

## Abstract

Replicative capacity of a cell is strongly correlated with telomere length regulation. Aberrant lengthening or reduction in the length of telomeres can lead to health anomalies, such as cancer or premature aging. Telomerase is a master regulator for maintaining replicative potential in most eukaryotic cells. It does so by controlling telomere length at chromosome ends. Akin to cancer cells, most single-cell eukaryotic pathogens are highly proliferative and require persistent telomerase activity to maintain constant length of telomere and propagation within their host. Although telomerase is key to unlimited cellular proliferation in both cases, not much was known about the role of telomerase in human parasites (malaria, *Trypanosoma*, etc.) until recently. Since telomerase regulation is mediated via its own structural components, interactions with catalytic reverse transcriptase and several factors that can recruit and assemble telomerase to telomeres in a cell cycle-dependent manner, we compare and discuss here recent findings in telomerase biology in cancer, aging and parasitic diseases to give a broader perspective of telomerase function in human diseases.

## 1. Introduction

The enzymatic repair of the loss of chromosome termini known as ”telomeres” by the RNA-protein enzyme ”telomerase” is an error-prone but highly evolved process that is critical for maintaining genome integrity. Two major problems arose with the emergence of linear chromosome as the widespread genetic material in the early phase of eukaryotic evolution [1] that could threaten the genomic integrity of eukaryotic cells. First, the cellular DNA polymerase, which uses short RNA primers to initiate DNA synthesis, was unable to complete the replication at the lagging strand termini of linear DNA [2]. This phenomenon is widely known as an “end-replication” problem [2]. These un-replicated ends can be mistaken for damaged/broken DNA and targeted to DNA repair pathway that restores broken chromosomes. This would ultimately result in the loss of genetic information as the cells will eventually stall their cell cycle in an attempt to repair the chromosomal end [3]. Additionally, there is always a constant threat of endonuclease activity at the un-replicated chromosomal ends. This is called an ”end-protection problem“. Clearly eukaryotic cells needed some special mechanism to protect their chromosomal ends and prevent DNA shortening. The solution to this “end-replication problem” came with the discovery of telomerase enzyme in the late 1980 [4,5]. Both in vitro and in vivo, telomerase enzyme activity was found to increase the length of chromosomal termini, the ”telomeres” by addition of repetitive DNA sequences using RNA as a template [4,5,6]. Furthermore, these custom-made, species specific DNA repeats act as a binding site for sequence specific telomeric proteins, thus forming a protective cap around chromosome termini, shielding it from the proteins of DNA repair pathway [7].

Telomerase is a ribonucleoprotein (RNP) enzyme comprised of two essential core subunits: the telomerase RNA (TER) and telomerase reverse transcriptase protein (TERT). TERT assembles with the integral RNA template within TER to repeatedly synthesize tandem repeats of DNA at the telomeric end of the chromosome. Although the general mechanism of chromosomal end maintenance by telomerase remains fairly conserved throughout the eukaryotic lineage from flagellated protozoan to multicellular eukaryotes, it seems that other organisms like *Drosophila* have lost TERT and TER and instead evolved reterotransposon based mechanism to maintain their chromosomal termini [8].

In mammals, telomerase is mostly expressed and active in the germ line cells but not in somatic cells. Expression of telomerase in somatic cells may lead to cellular immortality and eventually cancer. Akin to cancer cells, constant activity of telomerase is required for rapid proliferation and survival of eukaryotic pathogens of early origin (e.g. malaria, *Trypanosoma*, *Leishmania,* etc.). However, the biochemical similarity in telomerase function between the above cell types of vastly different evolutionary origin is yet to be determined. Such functional analogy between cancer and human parasitic diseases should be further exploited to find new therapeutic avenues since it has already brought some success in recent years [9]. In some eukaryotic parasites like *Trypanosoma* it has been found that any variation in telomere length directly affects the parasite’s antigenicity [10], which is directly related to the pathology of the disease. Telomerase function has also been associated with ageing as loss in activity leads to tissue necrosis and deficient tissue regeneration [11]. Also, mutations of either TERT or TER in humans are linked to numerous diseases like dyskeratosis congenital, pulmonary fibrosis and aplastic anemia [12]. This review will describe the evolution and structure-function relationship of telomerase components and their roles in human diseases with emerging new information on telomerase in parasitic diseases.

## 2. Telomerase Origin

Telomerase is required to maintain genome integrity in many eukaryotic cells [1,4]. Given the importance of this enzyme in maintaining genome stability, an obvious question arises: how did telomerase components originate in eukaryotic evolution? Did it appear instantaneously in early eukaryotes when linear chromosomes started to make an appearance or were they coopted from some preexisting molecular mechanism to maintain the chromosomal ends? The former scenario does not appear to be plausible as it is highly unlikely that both components of telomerase i.e. the protein component and its RNA template originated concurrently. For the later situation, there ought to be some sort of mechanism present in prokaryotes that transmitted in early eukaryotes to maintain their chromosomal termini. The answer probably lies in the presence of T-loop structure found in present day telomeres that also play a critical role in telomere protection [1,13]. T-loops are double stranded looped structure where the single stranded 3′ telomere termini invade the duplex repeat array to form a displacement (D) loop with tandem telomeric repeats. T-loops in mammalian cells can block DNA repair from non-homologous end joining (NHEJ) pathway by hiding the chromosomal termini. It is thought that in earlier eukaryotes these T-loop have the 3′ overhang and were able to solve the end-replication problem in the similar way as the prokaryotes by employing recombination-dependent replication (RDR) to rescue the replication fork once it encountered any lesion during replication. The initial steps of RDR required the T-loop formation and because of this similarity it is likely that the initial linear chromosomes having two or more repeats at their ends are transformed into T-loop by RDR enzymes [13].

Alternatively, the emerging eukaryotes containing circular genome could have been invaded by the Group II introns (self-splicing elements) introducing tandem repeats in the DNA, which may have led to chromosome linearization. Group II introns are the precursors of spliceosomal introns and non-LTR retrotransposons which uses reverse splicing and reverse transcription to efficiently incorporate into specific sites of DNA molecule. These elements might have crowded the circular chromosome with Group II intron repeats and a double stranded break in one of these DNA repeats gave rise to linear chromosome that was stabilized by the T-loop like structure [1]. Later on, as the linear chromosomes started becoming more stable and prevalent, the early eukaryotes had to evolve a more intricate mechanism to maintain the chromosomal termini which may have resulted in the modern-day telomerase. The presence of Group II intron Reverse Transcriptase might have act as a predecessor for the TERT.

The TERT component of telomerase is highly conserved, having a central catalytic domain which is identical to the conventional Reverse Transcriptase (RT) found across both eukaryotes and prokaryotes [14,15,16]. Besides, appearance of TERT in early branching eukaryotes [17,18] supports the theory that telomerase originated as an RT, might have interacted with an ancient progenitor RNA which later evolved into modern telomerase RNPs with various indispensable and stably associated TER components. Sequence comparisons between the TERT and another RT show that TERT is most closely linked to RTs of non-Long Terminal Repeat (LTR) retrotransposons [15,19]. Both components engage an RNA template for reverse transcription but differs only in the presence of endonuclease activity. The non-LTR retrotransposons endonuclease domain enables them to target chromosome interior [20]. Interestingly, a group of eukaryotic retrotransposons different from non-LTR and LTR retrotransposons, called terminal Penelope like Elements (PLE), have RT which lacks an endonuclease activity [20]. These are present at the chromosomal ends and also contain shorter telomeric repeats at their 3′ end [21]. It is plausible that the ancient retrotransposons similar to these terminal PLEs might be the progenitor of TERT proteins.

Akin to TERT, a possible progenitor of TER could be a retrotransposon RNA intermediate that was bound to retrotransposons [22,23]. But unlike retrotransposons both TER and TERT are encoded by separate genes [24,25]. This kind of relationship is also found in separately encoding retrotransposons, Long and Short Interspersed Nuclear Elements (LINEs and SINEs, respectively). SINEs are also long noncoding RNAs that are separately transcribed and interact with LINEs RT for DNA synthesis [26]. Hence, it is conceivable that an ancient RNA component like that of SINEs might have interacted with an ancient RT similar to LINEs to give rise to telomerase. However, mere interactions between two different macromolecules cannot entirely explain the evolution of active telomerase as it would have required several other events and players to form a nascent telomere extending enzyme. This would include the development of different structural elements within the RNA to stabilize the binding of TERT and interactions with other proteins during the course of evolution. This may also probably explain why diverse species engage different pathways for telomerase biogenesis to form an active RNP complex as described later in this review.

One can always argue that why not just retain the simple T-loop mode rather than evolving a complex mechanism for maintaining the chromosomal termini. The possible answer to this is that switching to telomerase based system has more advantages. First of all, it can de novo synthesize a new telomere and secondly, it might have provided more regulatory opportunities by connecting the cells to different pathways. However, not all eukaryotes engage in telomerase based telomere maintenance. As mentioned already, dipteran insects do not use the telomerase-based mechanism as a solution to end replication problem. Surprisingly, some human cells can maintain their telomeres without engaging telomerase. Apparently, these cells utilize a recombination-mediated telomere maintenance mechanism referred to as alternative lengthening of telomeres (ALT) [27], which has also been detected in a subset of human tumors [28].

## 3. Telomerase Architecture and Biogenesis

### 3.1. Telomerase Reverse Transcriptase Protein (TERT)

**Figure 1:**
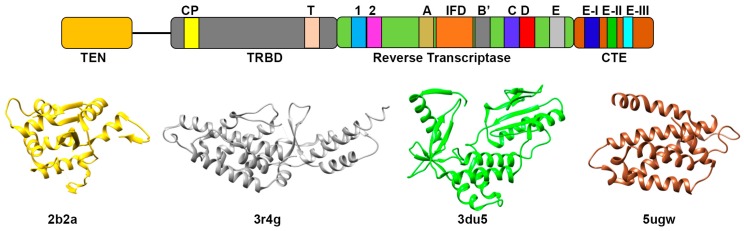
Schematic representation of TERT protein. Upper panel represents four domains of TERT protein as TEN, TRBD, Reverse Transcriptase and CTE domains. Motifs essential for activity of TERT protein domains are also shown in different colors. TEN and TRBD domains are joined by a linker. Lower panel shows TERT domains folding derived from crystal structures: Ten domain (PDB ID: 2b2a) and TRBD domain (PDB ID: 3r4g) from *Tetrahymena thermophila*, RT domain (PDB ID: 3du5) from *Tribolium castaneum* and CTE/thumb domain (PDB ID: 5ugw) from human.

As mentioned above, the minimal catalytic component of telomerase is composed of telomerase RNA (TER) and telomerase reverse transcriptase protein (TERT). TERT uses the RNA template to carry out de novo synthesis of telomeric DNA. Even though telomerase was discovered in late 1980s, it took almost 10 years to identify the first TERT subunits through yeast genetics [29] and biochemical purification of telomerase from *Euplotes aediculatus* [30]. Subsequently, TERT homologs were identified in humans, yeast, rodents, flagellates, *plasmodium* and ciliates [14,17,31,32,33,34]. From evolutionary perspective, TERT remains the most conserved subunit of telomerase. Universally, TERT encompasses four domains: Telomerase “Essential” N-terminal (TEN) domain, Telomerase RNA Binding Domain (TRBD), Reverse Transcriptase (RT) domain and C-Terminal Extension (CTE) or Thumb domain (Figure 1). The N-terminal TEN domain has a function of trapping the single stranded telomeric DNA and at the same time interacts with TER [35,36]. Since it binds to single stranded DNA it is likely that the TEN domain encourages processive repeat synthesis by capturing the substrate and retaining association with single stranded product [37]. Also, the crystal structure of *Tetrahymena* TEN domain has a mixed αβ fold (Figure 1) that helped in identification of conserved surface amino acid residues which were found to have key role in telomerase catalysis [38]. The TEN and TRBD domain are connected by an unstructured linker. TRBD domain is primarily an-all helical domain where the α helices are organized into two asymmetric halves that contains the residues of CP and TS motifs (Figure 1) [39]. The CP and TS motifs collectively form the TRBD RNA-binding pocket. Due to the chemical nature of the pocket it can bind to both single stranded and base paired RNA, probably Stem I and Template Boundary Element (TBE) of TER [39]. Reverse Transcriptase (RT) domain defines the catalytic/enzymatic domain of TERT. This is the most characterized and evolutionary conserved domain as it contains the seven conserved RT motifs essential for its activity [40] (Figure 1). RT domain consists of two subdomains which resembles the “finger” and ”palm” domains of classical RT enzymes [15,41]. The crystal structure of TERT from *Tribolium castaneum* shows that these regions are connected by loop which is involved in interactions with RNA-DNA hybrid (Figure 1) [42]. Another unique structural feature of the RT domain is the presence of large insertions between motifs A and B’, called ”insertions in fingers” (IFD) domain [14]. This domain is involved in protein-protein interaction that organize and stabilize the whole RT domain. Also, it was found that the TERT active site contains three aspartic acid residues which constitute a catalytic triad and is involved in metal dependent nucleotide addition [43]. Unlike RT domain, the CTE domain has low sequence conservation and may be involved in the species-specific functions. Structural comparison of *T. castaneum* TERT crystal structure with that of Human Immunodeficiency Virus (HIV) RT reveals that CTE adopts a distinctive protein fold representing thumb domain of telomerase. It is composed of α-helices connected by several loops which are involved in stabilization of RNA-DNA duplex [42,44]. The recent crystal structure of human CTE domain identifies three highly conserved regions within it namely motifs E-I, E-II and E-III (Figure 1) [45]. Mutations within these regions cause several human diseases like dyskeratosis congenital, aplastic anemia and idiopathic pulmonary fibrosis as they are shown to disrupt the binding between telomerase RT and DNA leading to the loss of telomerase activity and processivity [45].

**Figure 2:**
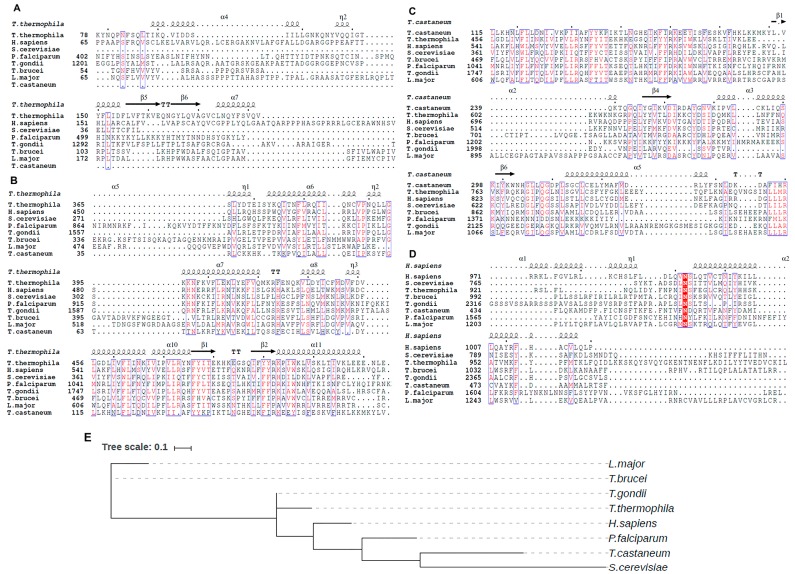
Sequence Alignment showing the conserved residues of TERT between *T. thermophila*, *H. sapiens*, *S. cerevisiae*, *P. falciparum*, *T. gondii*, *T. brucei*, *L. major*, *T. castaneum* for selected regions of (A) TEN domain (B) TRBD domain (C) RT domain and (D) CTE domain. The upper row in each alignment represents the secondary structure adopted by the domain in particular species where α-helix is represented as coil, β-sheet as arrows and β-turns as TT. Dots between the secondary structure elements or sequence alignment represent the deletion of residues/regions in species. Every 10th amino acid residue in the sequence is represented by a bold dot on the top. Red shaded regions represent the highly conserved residue across the species. (E) Phylogenetic tree between the TERT sequences of *T. thermophila*, *H. sapiens*, *S. cerevisiae*, *T. brucei*, *T. gondii*, *P. falciparum*, *T. castaneum*, *L. major*. *T. brucei* being ancestral eukaryote believed to have primitive TERT sequence from which TERT of *L. major* and other species evolved. Each species in the phylogenetic tree is connected to its branch through dashed line.

Comparative sequence analysis between the TERT protein of some selected species shows high sequence conservation in RT and TRBD domains while the TEN domain is less conserved and found to be absent in *T. castaneum* TERT (Figure 2A–C). A particular asparagine residue in CTE domain is found to be conserved in all the species (Figure 2D). This residue is a part of E-I motif which happens to be essential for nucleic acid binding. This was proposed because of the presence of large insertions. TERT from human parasites *Plasmodium falciparum* (malaria) and *Toxoplasma gondii* (toxoplasmosis) are longer than the TERT of other species. Phylogenetic analysis places TERT of *Trypanosoma brucei* as a putative progenitor from which TERT of other species might have evolved (Figure 2E). Surprisingly, even though the TERT from *P. falciparum* is larger in size, our phylogenetic analysis places it evolutionary closer to TERT of *T. castaneum* and *Saccharomyces cerevisiae*. As anticipated, TERT of *Leishmania major* was found to evolutionary similar to that of *T. brucei* (Figure 2E). 

### 3.2. Telomerase RNA (TER)

**Figure 3:**
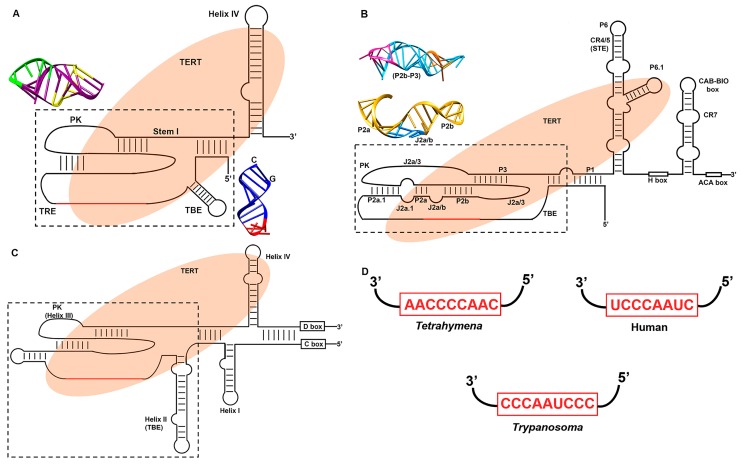
(**A**–**C**) Secondary structure of telomerase RNAs from *Tetrahymena*, human and *Trypanosoma brucei* NMR structure of TER domains are shown in colors. (**A**) Different domains of *Tetrahymena* TER are indicated as Template Boundary Element (TBE), Template Recognition Element (TRE), Pseudoknot (PK), Stem I and Stem Terminus Element Helix- IV. Also shown are: TBE (PDB ID: 2m22) with CG base pairing at stem terminus and red penta-nucleotide loop at apical position, triple helix PK (PDB ID: 5kmz) with turns in yellow and green. (**B**) In addition to above domains, human TER has long base paired PI, Stem Terminus Element CR4/5 and 3′ end with an H/ACA scaRNA domain comprised of H box, ACA box and in between CR7 stem loop having CAB box and BIO box at apical end. Also shown are NMR structure of domains: P2a-J2a/b (blue)-P2b (PDB ID: 2l3e) and triple helical P2b-P3 (PDB ID: 1ymo) with turns in magenta and orange of PK. (**C**) Secondary structure of *Trypanosoma brucei* telomerase RNA. Different domains are represented as Stem-loop Helix I, Template Boundary Element (TBE) as Helix II, Pseudoknot (PK) as Helix III, Stem Terminus Element as Helix IV and CD box domains at 5′ and 3′ end respectively. The template region is highlighted in red. Boxed area in TERs represent the catalytic core. TERT protein is shown as shaded region. (**D**) Sequence diversity between *Tetrahymena*, human and *Trypanosoma* TER template region are shown.

TER renders active site function providing RNA template which is required by the TERT for the de novo synthesis of DNA onto the chromosomal termini. In contrast to TERT, TER is the most versatile component of telomerase. It shows sequence and size divergence in wide range of eukaryotic species. Ciliates have the smallest reported TER, which is about ~150–200 nt long [46]. Vertebrate TERs range from ~310–560 nt [47] while yeast have the TERs ranging between ~780–1820 nt [48]. Although, sequence and secondary structure models of TERs from ciliates and higher eukaryotes were known for a while, it was not until recently TER components of deep branching parasitic microbes, which cause devastating impact on world health and economy, were described by our group and others [49,50,51]. The early branching eukaryotes like *Trypanosoma* have the TER of size ~1000 nt [49,50]. The largest TER of ~2.2 kb was reported from malarial parasite *Plasmodium falciparum* [51]. However, despite such size and sequence diversity, TERs from different species show broad evolutionary conservation in the mechanism of telomerase function. Figure 3A–C provides general reference to various TER domains described below.

#### 3.2.1. The Template

RNA template domain of telomerase provides sequence-specific regulatory features for telomerase action at the telomeres. It is a single stranded region of TER which is usually copied as ~1.5 repeats into telomeric DNA as a complementary sequence. It consists of two distinct segments: the 3′ alignment region which promotes base pairing with the DNA primer before commencing each cycle of DNA synthesis and the 5′ templating region specifying the nucleotide sequence that has to be synthesized. The alignment region of the template is vital for the telomerase Repeat Addition Processivity (RAP), a property of telomerase which allows repetitive synthesis of DNA repeats to occur at telomere without dissociating telomerase form the DNA strand [52]. Telomerase processivity at critically short telomeres are significantly elevated, which connects telomerase-mediated telomere maintenance to DNA damage response and repair pathways [53,54]. The length of alignment between TER and substrate DNA region is critical for the telomerase RAP. Telomerase of some rodent species have very low repeat addition processivity because the length of the alignment region is only 2 nucleotides [55]. In addition to influencing the RAP, the template specific sequence is also responsible for the regulation of telomerase enzymatic activity and the template utilization. Mutational analysis showed that the template is not just the carrier of sequence information as substitution of one or more nucleotides resulted in either slippage/misalignment of template or product dissociation [56,57]. Mutations in the human TER (hTER) template sequence alter the rate of telomeric repeat synthesis [58]. There is a signal nucleotide in hTER template, which acts as a pause site for the enzyme and hence defines the end of templating region. Mutations in this pause signal site results in aberrant telomeric DNA products [59].

#### 3.2.2. Template Boundary Element (TBE)

Template boundary element (TBE) demarcates the template region from its flanking non-template region at 5′ end. It prevents the telomerase from continuing DNA synthesis “overflow” into the non-template regions. Otherwise the resulting DNA sequences at chromosomal end will inhibit the binding of telomeric protein which is detrimental for telomerase function [60]. Till now, two different types of TBE have been identified for all known TER: (i) a template adjacent stem loop helix which is common in majority of eukaryotes and (ii) a long-range base paired core enclosing helix found only in vertebrates. The stem-loop helical structure is present at the 5′ end of template and is conserved in many eukaryotic species of ciliates, flagellates, fungi and echinodermal origin. It presumably works by restricting the availability of single stranded RNA by either TERT binding or by sequestering part of template residues base paired with TBE as evident from preliminary observations in *T. brucei* telomerase [61]. TBE from ciliates has a conserved sequence at a base of the stem that provides the binding site for CP motif located in TRBD domain of TERT [62,63]. In flagellates, TBE potentially can serve as TERT binding site [64]. TERT binding to TBE deter telomerase from utilizing the template-flanking sequence as template. In vertebrates, TBE is present in the form of core-enclosing helix, instead of a stem loop structure and functions as a tether that hinders the movement of TERT to the template-flanking region [65]. Remarkably, TER from some rodents lack a potent TBE and instead have only 2 nucleotides at the 5′ end of their template [66].

#### 3.2.3. Pseudoknot

A majority of the TERs have a unique pseudoknot fold present at the 3′ end adjacent to the template region. Pseudoknots of vertebrates and fungal TERs are larger, contain more stable helices and are indispensable for telomerase activity [24,67,68,69,70]. On the other hand, ciliate TER pseudoknot is rather smaller and less stable but important for in vivo telomerase function [57]. The pseudoknot from vertebrates and fungal TER binds to TERT protein and essential for telomerase enzymatic function [68,70]. Pseudoknot of hTER can assemble with TERT by itself in vitro, which can reconstitute its catalytic activity with RNA-DNA hybrid as a substrate [71]. A triple helix structure was detected within the helical junction of pseudoknot from hTER [72]. The presence of an invariant adenosine in this triple helix is essential for telomerase activity [73]. Also, a presence of sharp kink in the human pseudoknot structure at template proximal end is believed to be important for the template positioning and movements during the catalysis of chromosomal end [74,75].

#### 3.2.4. Stem Terminus Element (STE)

Apart from the template, TBE and Pseudoknot, every TER contains an additional motif that is found at the 3′ end of the template domain and is essential for TERT binding and its activity. This STE can occur as a terminal stem loop (Helix IV) in ciliates or flagellates [49,76], a stem loop emerging from a stem junction with bulged nucleotides (conserved region 4/5 (CR4/5) of vertebrates) [77] or a three-way junction as seen in budding yeast [78]. Even though the vertebrate STE is similar to that of budding yeast, the latter lacks the vertebrate specific stem loop P6.1 that is extremely well conserved. In contrast, the ciliate STE domain is only a stem-loop structure which lacks the three-way junction [79]. The exact mechanism by which the STE functions in telomerase catalytic activity remains mysterious. However considerable progress was made in determining the structure and binding position between CR4/5 and TERT protein [80,81]. Both ciliate and vertebrate STEs bind to TERT in vitro and greatly stimulate the TERT processivity [76,82,83]. UV-crosslinking experiments showed that CR4/5 binds mainly to the CP motif of TRBD [84]. One possible mechanism for STE function is that it regulates the correct positioning of RT-template docking by interacting with the TRBD and simultaneously delaying the active site closure by the TEN and the CTE domain until the template is properly placed [85]. Most current knowledge on STE function, based on studies in *S. pombe*, shows that deletion of STE impedes telomere maintenance, reduces normal levels of TER1 RNA and deters normal functioning of TBE [86].

### 3.3. Structural Variation in Telomerase RNA

#### 3.3.1. Ciliate Telomerase RNA Structure

TER from the ciliate *T. thermophila* is the smallest and simplest TER to be characterized so far. This TER was the first characterized one, is 159 nt long which includes all the conserved domains described above. The 9 nt template region (5′ CAACCCCAA 3′) is skirted at the 5′ end by TBE while the 3′ end is flanked by a pseudoknot. This whole complex is surrounded by a long-range base-pairing Stem I (Figure 3A). TBE, also known as Helix II in ciliate TER, is in the form of stem loop helix and is involved in the binding to TERT protein. The solution structure of TBE shows that the stem is formed by nucleotides involved in ~8 Watson-Crick base pairing and have a pentaloop cap at the apical end [87] (Figure 3A). The presence of conserved CG base pairing at the terminal end stabilizes the Helix II structure [87]. Nuclear Magnetic Resonance (NMR) structure of the *T. thermophila* pseudoknot shows that it is stabilized by the triple helix that includes both major groove and minor groove triple base pairing [88] (Figure 3A). Also, the telomerase pseudoknot analysis of *Tetrahymena* by Cryo Electron Microscopy (CryoEM) and NMR suggests that a pseudoknot may not form in the absence of TERT since a competing structure might form that can potentially sequester pseudoknot residues [88]. *T. thermophila* also contains an additional motif called template recognition element (TRE), which lies in between the template and pseudoknot. It presumably increases copying of telomeric repeats through mid-template positions and improves the usage of oligonucleotide templates in vitro [89]. RNA accordion model of TER suggests that TER is involved in the correct positioning of template in the active site and also template translocation which eventually increases the RAP of *Tetrahymena* telomerase [90].

The other ciliate *Paramecium* telomeric DNA consists of variable repeats of TTGGGG and TTTGGG. Closer inspection of *Paramecium* TER revealed that the sequence of template region and also the secondary structure is similar to TER of *T. thermophila* (Figure 3D) [91]. The mechanism of variable repeat synthesis of telomere by *Paramecium* TER is attributed to stuttering mechanism of RNP where during translocation the enzyme recedes by 1 nucleotide before completing the synthesis. This results in the addition of TTTGGG along with the conventional synthesis of TTGGGG [91].

#### 3.3.2. Human Telomerase RNA Structure

Human TER (hTER) is about 450 nt long which is an intermediate of ciliate and yeast TERs. In hTER and most vertebrates, the TBE is not in the form of stem loop helix but rather a long base-paired core termed as P1 stem which encloses the template and pseudoknot (Figure 3B). Pseudoknot of hTER, which is essential for activity is made up of stems and loops (P2a.1, P2a, P2b, P3, J2a.1, J2a/b, J2b/3 and J2a/3 respectively) (Figure 3B) [67]. Solution structure of hTER pseudoknot is similar to that of yeast and ciliate TERs consisting of triple helix (Figure 3B) [72]. Uridine rich loop J2a/b is extensively involved in base triples with adenine rich loop J2a/3, thus stabilizing the pseudoknot (Figure 3B). Examining the topology of the entire pseudoknot domain revealed that this J2a/b determines the overall shape of the core domain by inducing a large bend and hence bringing the P2 and P3 within 70 Å of each other [74]. This bend helps to bring the major groove of P3 and J2b-3 close enough to form a base triple as described above. The 3′ end consists of H/ACA, a small Cajal body-specific RNA (scaRNA) domain, including CR7 hairpin structure. H/ACA motif is in the form of hairpin-hinge-hairpin-tail secondary structure with H box is present at the hinge region and ACA box is located at tail end. CR7 comprising the hairpin loop have an essential Cajal body-specific localization signal (CAB box) and an additional unique biogenesis promoting (BIO) box which is also known to promote mature RNP accumulation in vivo [92].

#### 3.3.3. Flagellate Telomerase RNA Structure

Telomerase RNA of *T. brucei* is about ~900 nt long which is comparable to yeast but certainly larger than ciliates and vertebrates. The secondary structure model of *T. brucei* telomerase RNA determined by chemical probing of in vivo RNA [49] revealed that most of the residues of the *T. brucei* telomerase RNA (TbTER) template sequence of 5′ CCCUAACCC 3′ are modified suggesting that this template is available for strand specific hybridization to chromosomal termini (Figure 3D). This model also suggests the presence of a 5′ proximal stem loop called Helix I which is shorter than the yeast telomerase RNA (TLC1) that binds to Ku protein [93]. A long hairpin stem loop (Helix II) at the 5′ template proximal end is also present which can function as TBE (Figure 3C). In vitro RNA Selective 2’-Hydroxyl Acylation analyzed by Primer Extension (SHAPE) and mutational analysis of TbTER also identified a helix at the similar position, which was essential for telomerase activity providing evidence that this helix II can function as TBE [64]. Furthermore, akin to ciliate TBE, TbTER also has a CG pair at the base of TBE which stabilizes the Helix II structure in ciliate [87]. Even though no pseudoknot structure for TbTER was identified in in vitro SHAPE assay [64] possibility of having a pseudoknot structure through the presence of compensatory mutations can be identified through in vivo model [49] (Figure 3C). The 5′ and 3′ end of TbTER also contains the consensus sequences of C and D box, a hallmark of C/D snoRNA. Generation of computational model of TbTER revealed that the C/D box are partially paired to each other [50].

### 3.4. Telomerase Biogenesis and Maturation

While different TER domains described above are involved in binding to TERT and proper functioning of telomerase catalytic activity, TERs also possess some additional motifs which are involved in the RNA processing, RNP biogenesis and maturation inside the cell. These additional motifs can also act as binding site for other telomerase holoenzyme proteins which might regulate the physical interaction of telomerase with chromosomal ends. This section will deal with these specific-specific features that are involved in telomerase biogenesis in different species while their mechanism of action will be described elsewhere in this review.

#### 3.4.1. Ciliate Telomerase RNP Maturation

**Figure 4:**
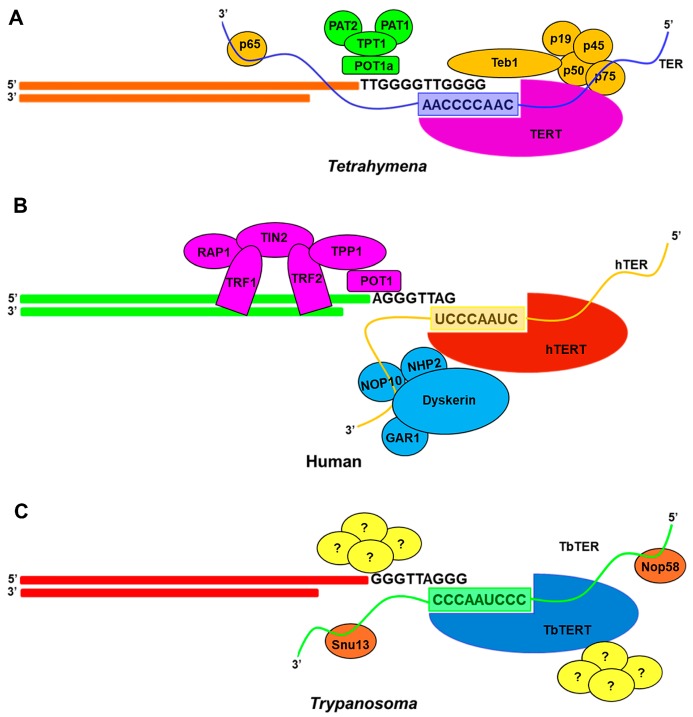
Telomerase and telomere associated protein are illustrated for (**A**) *Tetrahymena* (**B**) Human and (**C**) *Trypanosoma*. ‘?’ represents uncharacterized proteins involved in both the formation of active telomerase complex and associated to telomeric end in *Trypanosoma***.**

Ciliate TER is a unique RNA. Apart from being smallest among all TERs, it is also transcribed in a non-canonical way by RNA polymerase III [5]. RNA pol III adds polyuridine at the 3′ end which is retained by the TER after maturation of the primary transcript. In *Tetrahymena*, this polyuridine tail along with the TBE and STE binds to p65 protein (Figure 4A). This telomere specific protein is essential for TERT and TER accumulation in vivo which in turn generates a catalytically active RNP core [94,95]. Interactions with p65 induces and stabilizes a twist in the STE that is necessary for TERT binding and its catalytic activity [81,96,97].

#### 3.4.2. Yeast Telomerase RNP Maturation

Biogenesis of yeast telomerase RNP is more complex as compared to ciliate TERs. Studies are majorly focused on *Saccharomyces cerevisiae* TER, also known as Telomerase Component 1 (TLC1) and more recently on *Schizosaccharomyces pombe*. After being transcribed by RNA polymerase II, TERs accumulate mainly as non-polyadenylated transcripts while a small fraction of polyadenylated form is also present [98,99]. Only non-polyadenylated TER forms active telomerase holoenzyme complex [98,100].

After the formation of a primary transcript, the 3′ end of mature TLC1 is generated by Nrd1-nab3-Sen1 pathway proteins which are also involved in the maturation of small nuclear (sn) and small nucleolar (sno) RNAs transcript in yeast [101,102]. During transcription termination the TRAMP (Trf4, Air2, Mtr4) polyadenylation complex comes into play and adds a short polyA tail at the 3′ end, only to be removed later by nuclear exosome [101,103].

The processing pathway of *S. pombe* TER1 is different from *S. cerevisiae* TLC1. In *S. pombe*, TER1 is transcribed as a precursor containing an intron immediately downstream of its mature 3′ end. The exon-intron junction is recognized by the spliceosome; however, the spliceosome only performs the first cleavage reaction and releases the 5′ exon to become the active form of telomerase RNA [104]. Once mature, both TLC1 (*S. cerevisiae*) and TER1 (*S. pombe*) assembles with Sm proteins (SmB, SmD1, SmD2, SmD3, Sme, SmF and SmG) to promote RNP accumulation and also TER cap hypermethylation via 2,2,7-trimethylguanosine (TMG) synthase (Tgs1) [105]. After capping TLC1 is exported to cytoplasm where it is bound by TERT subunit Est2 followed by Est1, Est3 and Ku proteins [106,107]. In *S. pombe*, Lsm2–8 complex replaces the Sm ring at 3′ end. This switching not only protects the 3′ end of TER1 from exonuclease activity but also enhances its association with the TERT protein (Trt1), thus generating an active RNP complex [108].

#### 3.4.3. Human Telomerase RNP Maturation

The biogenesis pathway of human telomerase (hTER) is similar to the H/ACA box containing small nucleolar (sno) and small Cajal body (sca) RNAs. For maturation and biogenesis, hTER contains additional motifs both at the 5′ and 3′ end [109]. After the RNA is transcribed by RNA polymerase II, a preassembled H/ACA protein complex of NHP2, NOP10, dyskerin and H/ACA RNP chaperone NAF1 is loaded on the H/ACA motif at the 3′ end of the transcript [110,111,112] (Figure 4B). The precursor is then processed at the 3′ end up to the boundary of ACA motif by an unknown mechanism. The 5′ end of this TER has several guanosine residues which increases the mature RNA buildup by forming a G-quadruplex that is unfolded by helicase DHX36 [109].

After the assembly and processing, the hTER is then imported into Cajal bodies by the transportation factor Phosphorylated Adaptor RNA Export protein (PHAX) and Nopp140 [113,114]. Here the hTER gets its TMG cap at the 5′ end by Tgs1, the methyltransferase enzyme responsible for TMG synthesis [115,116]. Also, the Cajal Body (CAB Box) associated proteins TCAB1/WDR79 helps in concentrating the mature RNP inside the Cajal body [117,118]. This association is extremely important for telomere maintenance as mutations in TCAB1/WDR79 are linked to several human diseases like dyskeratosis congenita [118]. Binding of hTERT to hTER is not required for its biogenesis but the former could have a stimulatory effect on latter when overexpressed [119]. Both hTER and hTERT are present in the nucleoli during the early G0-phase of the cell cycle but they do not share the same compartment. hTER is mainly present in the Cajal bodies which is present at the periphery of the nucleolus while hTERT is primarily concentrated at nucleoloar foci but in the early S-phase hTERT is found to be physically associated with the Cajal bodies containing hTER [120]. It remains a mystery how the co-localization of both hTER and hTERT is coordinated from different nucleolar compartments to form an active RNP complex [121]. However, the assembly of hTER to hTERT is a highly chaperoned process and thought to be mediated by Hsp90 as geldanamycin, an inhibitor of Hsp90 is found to decrease the active human telomerase RNP production and initiates proteolysis of hTERT [122,123,124]. Other than Hsp90 there are other proteins like DNA dependent protein kinase interacting protein, KIP and snRNP assembly factor Survival of Motor Neuron (SMN) which may be involved in organization of active human telomerase complex [125,126]. 

#### 3.4.4. Flagellates Telomerase RNP Maturation

Even though flagellate and ciliates are phylogenetically related and ciliates are thought to have evolved from flagellate ancestors [127], they do not share many common features of telomerase and its biogenesis process. Our current knowledge of telomerase and telomere biology in flagellates is mostly derived from recent findings in *Trypanosoma* sp. [49,50] and *Leishmania* sp. [128]. So far, little is known about the biogenesis of the TERT component in these flagellates species. However, two recent publications on characterization of *T. brucei* revealed that the telomerase RNA transcript is transcribed by RNA polymerase II and is processed by trans-splicing mechanism for maturation [49,50]. This spliced leader sequence in TER interact with methyltransferase-associated protein (MTAP) which is equivalent to TCAB1 protein of vertebrates [50]. Secondly, Gupta et al. [50] also proposed that *T. brucei* TER (TbTER) contains a novel C/D box domain, instead of the typical H/ACA box found in the 3′ end of vertebrate TERs. This observation was based on the evidence that *T. brucei* TER binds to the box C/D proteins Nop58 and Snu13 [50,129] (Figure 4C) and thus biogenesis of TER in kinetoplastid flagellates could be different from that of vertebrates. TbTER contains a splice-leader RNA (SL-RNA) which is essential for maturation via trans-splicing but it seems to lose SL while associating with *T. brucei* Telomerase Reverse Transcriptase (TbTERT) [49]. However, the exact mechanism by which an active RNP complex is assembled in flagellates is still unknown and will require more extensive in vivo and in vitro studies to determine the same.

## 4. Recruitment of Telomerase RNPs to Telomere

### 4.1. Coupling of Ciliate RNP to Telomere

TERs of *Tetrahymena* and *Oxytricha* are known to localize in numerous spherical foci of macronucleus which does not contain any DNA or telomeric protein [130,131]. Recruitment of the ciliate telomerase RNP complex to its substrate is mediated by the binding of five proteins designated as p19, p45, p50, p75 and Teb1. Proteins p19, p45, p50 and p75 first constitute to form a telomere adaptor sub-complex (TASC) which finally recruit Teb1 [132] (Figure 4A), a single stranded telomeric DNA-binding protein. Teb1 has an oligonucleotide/oligosaccharide (OB) fold architecture similar to large subunit of Replication Protein A (RPA). This fold helps Teb1 protein to bind ~15 nt single stranded (ss) telomere with high specificity and affinity [133]. The binding of *T. thermophila* telomerase to its substrate may also require the involvement of telomere associated protein Pat1, depletion of which could cause telomere shortening [134].

### 4.2. Coupling of Yeast RNP to Telomere

After the biogenesis of *S. cerevisiae* TLC1 in nucleolus, it is exported to cytoplasm where the TER binds to various protein components along with TERT before it is imported back into nucleus. *S. cerevisiae* telomerase RNP is linked to telomere via Cdc13 which is also high affinity, telomere specific, single-stranded DNA (ssDNA) binding protein [135,136]. Cdc13 can either interacts with Stn1-Ten1 to form telomere capping CST complex [137] or can bind to Est1 and recruit telomerase RNP to chromosomal termini [138]. Also, Ku proteins, which play key role in recognition and repair of double stranded DNA breaks have been anticipated to play a role in telomerase recruitment to the substrate. This could be done in two ways. Ku proteins can boost the nuclear import of TLC1 RNPs [139]. Alternatively, some studies have shown them to act as a bridge between TLC1 and telomeric DNA [140]. 

Recruitment of active telomerase RNP complex in *S. pombe* to its substrate is different from *S. cerevisiae*. In *S. pombe* the TER1 bound Est1 interacts with coiled-coil protein quantitatively enriched 1 (Ccq1), which is already bound to ssDNA through Pot1-Tpz1 complex. This association recruits the whole RNP to the ssDNA end [141,142].

### 4.3. Coupling of Human RNP to Telomere

As discussed above both hTERT and hTER are initially localized in different nucleolar compartments. But at the start of S-phase hTERT is found to be co-localized with Cajal bodies containing hTER. Coupling of human telomerase to telomere requires TIN2 and TPP1 telomere proteins [143,144], which are already docked to the double stranded region of telomere through TRF1 and TRF2 [145,146]. Akin to ciliate Teb1, TPP1 has an OB fold which could bind to TEN domain of TERT protein [147]. TPP1 is also found to interact with single stranded region of telomeric DNA via POT1 (Figure 4B). This POT1-TPP1 complex is essential for human telomerase activity as it is known to maintain the association of RNP with DNA during template translocation [148]. Like yeast, human Ku heterodimer could interact with hTER and/or hTERT [149]. Ku70 in human interacts with TRF2 to concentrate Ku to telomeres and thereby preventing telomere loss [150].

### 4.4. Coupling of Flagellates RNP to Telomere

TbTER co-localizes with TbTERT outside the nucleolus [50] but how the mature telomerase RNP complex of flagellates is recruited to its single stranded telomere is still an unanswered question. Studies involving UV-crosslinking followed by Mass-Spectroscopy (MS) analysis in *T. brucei* extracts have detected the presence of protein complexes which have high affinity for G-overhangs and share similar characteristics with Est1 protein and other G-rich single stranded telomere end-binding proteins of yeast, human and ciliate [151]. However, no known protein homologs of the above complexes have been detected so far raising the possibility that proteins involved might be novel to *T. brucei* [151], which still remain to be characterized (Figure 4C).

## 5. Factors Involved in Telomerase Assembly and Activity

Telomerase activity can be modulated by its associated proteins that are involved in RNA maturation, telomerase assembly, telomerase recruitment and telomere binding. The telomerase core ribonucleoprotein (RNP) has a telomerase reverse transcriptase (TERT) catalytic protein subunit and an intrinsic telomerase RNA (TER) that provides the template for telomere DNA synthesis. Telomerase also often interacts with species-specific factors: in humans, Hsp90, p23 and TER binding proteins, L22 and human Staufen (hStau), are associated with telomerase activity [152]. Dyskerin, Nucleolar protein family A member 2 (NHP2) and NOP10 bind the H/ACA snoRNA motif of mammalian TER [110] and stabilize newly transcribed TER RNA, which is required for proper localization of telomerase. Dyskerin is one of the evolutionary conserved components of telomerase RNP complex as AtNAP57, the *Arabidopsis* dyskerin is also required for maintaining the *Arabidopsis* telomere [153]. In *S. cerevisiae*, Sm proteins are necessary for telomerase RNA stability, while three other proteins, Est1, Est3 and Cdc13, are dispensable for in vitro telomerase activity but are required for telomere length maintenance [154]. In *S. pombe*, sequential binding of Sm and Lsm proteins to TER is important for TER maturation [108].

In addition to telomerase-specific factors, telomere-binding proteins play pivotal roles in telomerase activity regulation [155]. A six-protein ”shelterin” complex has several important contributions to this process controlling telomere length maintenance and telomerase activity. These proteins are: telomere repeat binding factors 1 and 2 (TRFs), repressor/activator protein 1 (RAP1, most evolutionary conserved shelterin, deficiency of which leads to increased telomere recombination), protection of telomeres 1 (POT1), TRF1 interacting nuclear factor 2 (TIN2) and TPP1 (Figure 4B). It is known that telomeric protein POT1 directly interacts with the 3′ end of telomere and binds TPP1 [147,156,157]. This POT1-TPP1 inhibits telomerase access to telomeres and provides a physical link between telomerase and the shelterin complex [147,148,156]. Mutations identified in POT1–TPP1 complex are suspected to be associated with human diseases, such as familial glioma, melanoma and chronic lymphocytic leukemia [158].

## 6. Pathophysiology of Telomerase

For maintenance of the chromosome end structure and hence chromosome integrity, regulation of telomerase activity is necessary. However, several studies have demonstrated that any deviation from the regulated process involving telomerase and telomere preservation is linked to variety of human diseases ranging from cancers to age related disorders. Telomere binding proteins are also known to participate in disease progression caused by human parasites. In this section, we will briefly describe the association between telomerase and various diseases along with the necessity to understand the telomerase structural and functional characteristics for the development of telomerase targeted therapies.

### 6.1. Ageing, Cellular Immortality and Cancer

Telomere elongation in human is a cell-cycle regulated process [159]. Telomere is elongated by telomerase during the S-phase until it enters into the M-phase for cell division [160]. Human germ line and stem cells that proliferate indefinitely and hence immortal have active telomerase which maintains the telomere length throughout the cell division [161]. Conversely, in most of the differentiated somatic cells that have reduced life span, telomerase is found to be inactive and hence these cells are called mortal cells. In these cells, the telomere length decreases with each cell division as opposed to the immortal one and thus establishing the connection between telomerase, chromosomal stability and mortality of cells. Thus, telomere shortening and inactive/repressed telomerase in normal somatic cell may be indicative of some kind of ‘telomere clock mechanism’ that elicits cellular senescence [162], loss of proliferative capacity and ageing. The exact mechanism of telomere attrition is still obscure but it is thought that this may be due to the loss of TRF2 from the chromosomal termini. Studies have shown that TRF2 can promote the formation of T-loop (described above) in vitro and may be in vivo as well [163]. Due to the loss of TRF2 the chromosomal termini continue to shorten, as they are unable to form T-loop. The exposed chromosomal end can then activate the p53 or Ataxia-Telangiectasia Mutated (ATM) kinase mediated cell apoptosis pathway [163]. In the absence of telomerase activity, telomeres will shorten with each round of cell division until they reach a short length threshold known as “the Hayflick limit” [162]. Once they reached this threshold, p53 and pRB dependent DNA damage response pathway is activated hence eliciting the non-proliferative state called replicative senescence [164,165]. This state impedes the onset of tumorigenesis but cells with defective p53 and pRB (retinoblastoma) pathways have the ability to evade this barrier and can undergo additional cell division [166]. However, during the period of additional cell division the telomeres will continue to shorten and ultimately have to undergo the breakage-fusion-bridge (chromosome end fusion) cycle which stimulates genomic instability and hence activates second proliferative blockage which is followed by cellular apoptosis [167,168]. Sometimes, the cell can also evade this second barrier by either up-regulating the telomerase activity or by alternative telomere lengthening (ALT) mechanism which can stabilize the short telomere and allow the cells to divide again [169]. This give rise to cellular immortality and eventually could lead to tumorigenesis. Most of the human cancer cells (80–90%) constantly express telomerase catalytic subunit and employ telomerase dependent telomere elongation mechanism to achieve the infinite growth potential, which make activation of telomerase holoenzyme as one of the most common pathway for cellular immortalization [170]. The rest of the tumor cells use ALT pathway, which involves homologous recombination based for telomere maintenance [169]. A better understanding of this ALT based pathway is now necessary to develop new strategies for combating cancer. As discussed above, telomere attrition results in aging and cellular senescence.

Mechanism of telomerase activation in cancerous cells has been studied extensively. Among series of mutations that are identified in key regulatory regions, mutations in the promoter region of *hTERT* in most of the tumor cells are deemed important, since these mutations could be an early event in the onset of carcinogenesis [171]. *hTERT* is a 40 kb long gene comprising of 16 exons and 15 introns and have a promoter region which is not only GC rich but also lacks both TATA and CAAT regions. Recent studies have shown the presence of two exceedingly frequent mutations, C→T at −124 bp and −146 bp upstream from ATG start site. These mutations, thus generates an E-twenty-six (ETS) transcription factor binding site which can up-regulate the *hTERT* expression in cancerous cells [172,173]. One study reported that epidermal growth factor (EGF) mediated activation of telomerase activity in lung cancer is linked to the binding of ETS-2 to *hTERT* promoter [174]. Furthermore, some less frequent *hTERT* mutations resulting in A→C and/or C→A transitions are also reported in some other cancer lines [175]. Although these promoter mutations are being most frequent in cancer, their level and frequency differs with cancer types. Melanoma, skin carcinoma and liver cancer have highest frequencies of TERT promoter mutations while gastric cancer, pancreatic cancer and gastrointestinal stromal cancer reported to have lower frequency of these mutations [173,176,177]. In prostate cancer cells, expression of both *hTER* and *hTERT* are found to be directly correlated to the expression of myelocytomatosis viral oncogene (MYC) as silencing of the later results in decreased expression of *hTER* thus causing reduced cell proliferation [178]. The exact mechanism of this correlation is still under investigation but it is thought that mutations in the *hTERT* promoter enhances the binding of MYC via the increased binding of ETS/T-cell factor (TCF) [179]. Nevertheless, highly activated telomerase becomes an appealing target for the development of novel anti-cancer therapeutics [28,169].

### 6.2. Dyskeratosis Congenita: Case of Telomerase Dysfunction

As opposed to the cancer where telomerase is found to be hyper active there are certain other telomerase related human diseases where telomerase activity was found to be wanting. Dyskeratosis congenita appears during the early stage of human life and is characterized by three major symptoms: dystrophy of nails, hyperpigmentation of skin and oral leukoplakia. Additional symptoms include: developmental delay, organ failure and premature hair loss. A major cause of mortality among the patients is due to bone marrow failure and pulmonary fibrosis [180]. The hallmark of this disease is the presence of short telomere [181] in the cells which arises due to the mutations in the genes encoding either the telomerase or the component of telomerase holoenzyme. Mutations of *DKC1* which encodes dyskerin, TRF1-interacting nuclear factor (*TINF2*) encoding shelterin component TIN2, genes encoding TERT and TER accounts for most of the dyskeratosis congenita cases [181,182]. Less frequent mutations in *NHP2*, *NOP10* and *TCAB1* have also been reported to help in disease onset [118,183,184]. Dyskerin, NHP2, NOP10 and TCAB1 are important for human telomerase biogenesis and mutations thereof decrease the protein stability and hence its activity [181,185]. As stated earlier, mutations in the C-terminal extension of TERT protein is responsible for various bone marrow failure syndromes [45]. These mutations disrupt the binding of protein to its substrate leading to incomplete telomere extension and telomere attrition while the overall stability or the expression of the TERT protein remains unaffected. Telomere attrition is also linked to severe liver complications in patients with bone marrow transplant [186] and heart diseases [187]. It is important to understand the fundamental mechanism of diseases associated with telomere attrition as there are no known available treatment for associated diseases at present except for the organ transplant [188]. One possibility is that due to the repeated shortening, telomere can no longer binds to the shelterin complex. These short telomeres can then activate the DNA damage response similar to the one resembling DNA double stranded breaks [165]. The following signaling cascade can then induce cell senescence or apoptosis and in some cases, both can take place concurrently [189].

### 6.3. Telomere, Telomerase and Human Pathogens

One of the strategies which make several eukaryotic pathogens effectively virulent and pathogenic is antigenic variation. Because of the mechanism of antigenic variation, pathogens are able to evade the host immune mechanism and can live inside their host for long time. Major classes of virulence related genes in these pathogens are present in close proximity to telomeres, underscoring the role of telomere maintenance pathways in integrity and activity of these genes.

#### 6.3.1. Telomere, Telomerase and Virulence in *Trypanosoma brucei*

*T. brucei* is an extracellular human parasite which causes a vector borne disease, called African trypanosomiasis. It is a highly successful pathogen as it can effectively evade the human immune system and can live inside the human host persistently, thus allowing chronic infection. The success is due to the unique composition of the variable antigens expressed on pathogen cell surface, which is composed of 10^7^ copies of compactly filled single Variable Surface Glycoprotein (VSG) [190]. The interesting feature of VSG is that it is highly immunogenic but the pathogen is still able to evade the human immune response by switching the expression of one *VSG* gene to another [191] within a short period of time. This switching of surface antigen expression is possible due to the presence of more than 1000 highly divergent *VSG* genes located at the sub-telomeric region of the *T. brucei* chromosomes [192]. The sub-telomeric regions where the VSG lies along with the telomeres are usually unstable and it is thought that telomere associated proteins play a role in the switching of antigenic genes by either duplicative Gene Conversion (GC) mechanism or by reciprocal Telomeric Exchange (TE). In later, a new VSG replaces the previously active VSG at expression site through homologous recombination event [193], whereas GC involve the non-reciprocal transfer of VSG at expression site as the previously active VSG is lost from genome [194]. The proximity of VSG to telomeres make them an excellent candidate for GC event through Double-Stranded Break (DSB) [195]. Increased level of VSG switching frequency was observed in the *TbTERT*-null cells because of the presence of extremely short telomere [10]. It is proposed that in *T. brucei* the presence of short telomere frequently favors chromosomal end breakage hence coercing the pathogen to undertake antigenic variation by damaging the active VSG [196]. Additionally, one of the central component of *T. brucei* telomere component *T. brucei* repressor/activator protein 1 (TbRAP1) is known to regulate the VSG expression by silencing the genes encoding them [197]. Furthermore, this regulation is more prevalently close to telomere than at upstream end suggesting that telomere structure is critical for silencing. Surprisingly, the pathogen has the ability to maintain the short telomeres without any significant problem indicating existence of an alternative telomerase independent telomere maintenance mechanism in *T. brucei* [196]. This arises an interesting question that if the short telomeres are important for pathogen viability then why did *T. brucei* evolve an intricate RNP complex like telomerase? Further studies on TbTER and TbTERT are indeed necessary to understand the underlying mechanism of telomerase regulation in trypanosomes.

#### 6.3.2. Telomere, Telomerase and Virulence in *Plasmodium falciparum*

Malaria remains as one of the deadliest human disease. It is a mosquito borne infectious disease caused by the intracellular protozoa *Plasmodium* that infect millions of people worldwide every year. There are five different species of *Plasmodium* parasite of which *P. falciparum* is most lethal because of its high mortality and morbidity rate. Currently there is no effective vaccine available and the most concerning fact is the emergence of multidrug resistance strains of the parasite. *P. falciparum* have 14 linear chromosomes of varying length [198]. The telomere of each chromosome is preceded by 15–30 kb sub-telomeric region called as telomere associated sequences (TAS) [199]. TAS of each chromosome are highly organized and are made of 6 blocks of telomere associated repeat elements (TAREs 1–6) [199]. Upstream of these sub-telomeric regions are the hotspots of genes which code for the essential virulence factors called *var* and *rifin* genes in *P. falciparum* and *vir* genes in *P. vivax* [200,201]. The mechanism by which these *var* genes are activated and switched are still under active investigation but the proximity of these genes to telomere does make telomere biology much intriguing. One theory is the presence of telomere associated silent information regulator 2 (sir2) protein at the sub-telomeric region of *Plasmodium* genome which is known to maintain the heterochromatin structure [202,203]. Pfsir2 is a histone deacetlyase which is already known to cause histone acetylation and thus gene activation and the reverse is true for gene repression [204,205]. It is plausible that the binding of Pfsir2 to the upstream promoter region of *var* genes may silence them and thus regulates the expression of sub-telomeric the antigenic genes [203]. Interestingly, deficiency of Sir2A triggers abnormal lengthening of telomeres in *P. falciparum*, however if and how this unusual lengthening of telomere and virulence gene regulation is linked to telomerase mediated telomere length homeostasis is still unknown.

In *P. falciparum*, the mean telomeric length (~1.2 kb) is maintained constantly even during the highly proliferative stages of the parasite life cycle like the bloodstream stages [199]. As discussed above maintenance of the telomere is important for the virulence of the pathogen. Apart from maintaining the telomere, telomerase is also involved in repairing the broken chromosomal end. This particular RNP has some unique features. TERT component of telomerase is ~3 times that of its yeast homologue [34] while its RNA component is also known to be the largest TER [51] and like TERT [206], is essential for survival of the parasite [207]. *P. falciparum* TERT is a basic protein because of the presence of several stretches of asparagine and has the molecular mass of ~280 kDa and contains all the motifs specific to the known TERTs [34]. While the biogenesis of *P. falciparum* telomerase is still not clear, it is known that *P. falciparum* Telomerase Reverse Transcriptase (PfTERT) is mainly localized in the nucleolus [34]. However, further studies are necessary to better understand the biogenesis of telomerase and elongation of telomeres in *P. falciparum* and its role, if any in the disease progression and pathogenesis.

## 7. Conclusions and Future Direction

Over the course of eukaryotic evolution telomerase originated as a primary solution to the chromosomal ‘”end-replication problem” and to shield the chromosomal termini from being recognized as a substrate of DNA repair pathway. Telomerase is an intricate RNP complex comprising of a long non-coding, structural RNA associated with a protein containing reverse transcriptase activity. While telomerase from different species are highly varied in their structure, process of biogenesis and maturation, there are some universal features of telomerase which are shared between various species. Telomerase and telomere biology have been extensively studied in human and yeast but the same in not true for clinically important eukaryotic pathogens like *Plasmodium*, *Trypanosoma*, *Toxoplasma*, *Pneumocystis* etc. Numerous studies have shown the effect of telomere on the pathogenesis and virulence of these pathogens which harbors different virulence genes near the telomeres. To this end, it is still a mystery how the expression of these genes is coordinated with the telomere length homeostasis process, which is primarily coordinated by telomerase. One hypothesis is that these genes are regulated through Telomere Position Effect (TPE). TPE was first discovered in *Drosophila melanogaster* [208,209] and later on was extensively studied in yeast [210,211]. TPE is a biologically prevalent phenomenon which involve silencing of the genes present in the proximity of telomeres and more specifically in the heterochromatins, thus making it as a global mode of gene regulation. There is compelling evidence in yeast showing that the TPE is dependent on telomeric length and increasing the telomeres improves TPE [212,213,214]. Besides, TPE was also found as regulator of genes adjacent to human telomeres [215] and overexpression of both *hTERT* and *TRF1* regulate TPE [215,216]. All together these studies along with other shows that telomere length and architecture have direct or indirect effect on the regulation of sub-telomeric genes and cross-talks with telomerase activity and regulation. Therefore, extensive biophysical and biochemical studies on telomerase holoenzyme is further required to understand its precise role in telomere biology. In addition, these studies will also open a new boulevard for the development of novel therapeutics which could be aimed in improving the diagnosis and treatment of numerous human diseases associated with either telomerase dysfunction or telomeric silencing.

## Abbreviations

RNP	Ribonucleoprotein
TER	Telomerase RNA
TERT	Telomerase Reverse Transcriptase
NHEJ	Non-Homologous End Joining
RDR	Recombination-Dependent Replication
PLE	Penelope Like Elements
ALT	Alternative Lengthening of Telomerase
TEN	Telomerase “Essential” N-terminal
TRBD	Telomerase Binding Domain
RT	Reverse Transcriptase
CTE	C-terminal Extension
RAP	Repeat Addition Processivity
TBE	Template Boundary Element
PK	Pseudoknot
STE	Stem Terminus Element
CR4/5	Conserved Region4/5
snRNA	Small Nuclear RNA
snoRNA	Small Nucleolar RNA
CAB	Cajal Body
scaRNA	Small Cajal Body RNA
TASC	Telomere Adaptor Sub-Complex
RPA	Replication Protein A
VSG	Variable Surface Glycoprotein
GC	Gene Conversion
TE	Telomeric Exchange
DSB	Double Stranded Break
TAS	Telomere Associated Sequences
TAREs	Telomere Associated Repeat Elements
TPE	Telomere Position Effect
